# Patient-specific distal radius locking plate for fixation and accurate 3D positioning in corrective osteotomy

**DOI:** 10.1007/s11751-014-0203-1

**Published:** 2014-11-02

**Authors:** J. G. G. Dobbe, J. C. Vroemen, S. D. Strackee, G. J. Streekstra

**Affiliations:** 1 Medical Imaging Section, Department of Biomedical Engineering and Physics, Academic Medical Center, University of Amsterdam, Room No. L0-113-3, Meibergdreef 9, 1105 AZ Amsterdam, The Netherlands; 2 Department of Plastic, Reconstructive and Hand Surgery, Academic Medical Center, University of Amsterdam, Amsterdam, The Netherlands

**Keywords:** Additive manufacturing, Corrective osteotomy, Computer-assisted surgery, Virtual planning

## Abstract

Preoperative three-dimensional planning methods have been described extensively. However, transferring the virtual plan to the patient is often challenging. In this report, we describe the management of a severely malunited distal radius fracture using a patient-specific plate for accurate spatial positioning and fixation. Twenty months postoperatively the patient shows almost painless reconstruction and a nearly normal range of motion.

## Introduction

Malunion following a distal radius fracture is a common complication treated by osteotomy surgery. Accurate reconstruction is important since a statistically significant relationship has been found between malpositioning and clinical outcome [1]. It has been shown that standard anatomical plates may lead to considerable positioning errors in individual patients [2]. Three-dimensional (3D) techniques are increasingly valuable for preoperative osteotomy planning [3–8]. However, implementing the preoperative plan into the patient is not a trivial task and may require complex navigation techniques [3, 5] in order to assure accurate bone positioning in 3D space. Techniques have been described that use guides for cutting and for temporary bone alignment [6, 7]. Positioning, however, may deteriorate during application of standard osteosynthesis material.

In this report, we describe the successful management of a severely malunited distal radius fracture using a novel patient-specific plate, which fits the bone geometry and accurately restores bone alignment.

## Case report

**Fig. 1:**
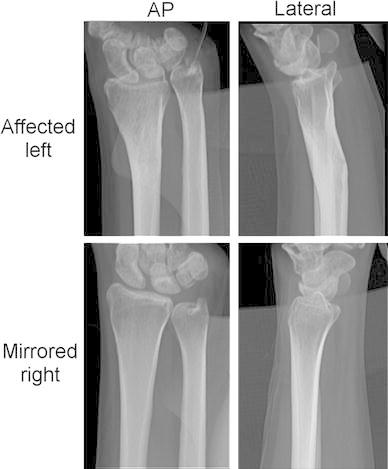
Anteroposterior and lateral radiographs of the affected wrist (*top row*) revealing a malunion of the radius and of the mirrored healthy wrist (*bottom row*)

A 40-year-old right-handed woman sustained a distal radius fracture during childhood. Initially, the left forearm fracture was treated by standard repositioning and cast application in a hospital elsewhere. The patient presented at our institution more than 30 years after the initial injury. She reported chronic pain and restrictions in daily activities due to reduced wrist function. She used pain medication on a daily basis. Radiographic evaluation (Fig. 1) confirmed a malunion of the distal radius.

Physical examination showed a left wrist extension and flexion of 30° and 10°. Radial and ulnar deviation were 15° and 35°. Forearm supination and pronation were both 45°. Grip strength and sensation were intact.

Approval of the institutional review board was waived, and informed consent of the patient was obtained.

### Surgical planning

**Fig. 2:**
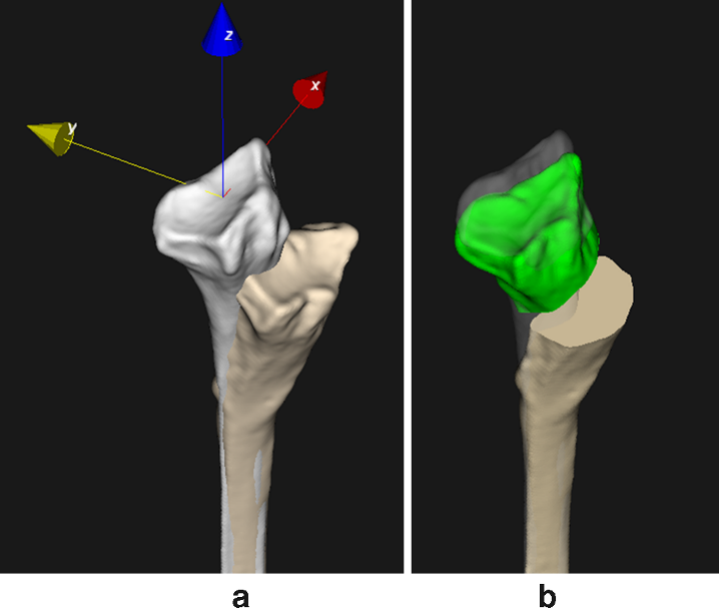
**a** Mirrored healthy radius (*white*) proximally aligned with the affected left radius. The affected radius is markedly shortened, shows a rotation deformity, and shows radial and dorsal collapse. The anatomical coordinate system is used to quantify the deformity. **b** Planned position of the distal radius (*green*) based on the contralateral side and corrected for bilateral length discrepancy

**Fig. 3:**
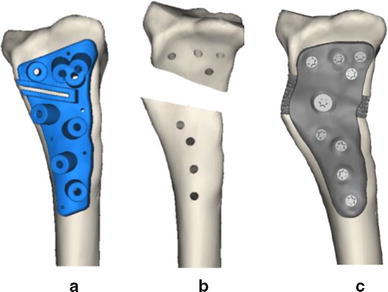
Simulation of surgical treatment. **a** Affected bone with drilling and cutting guide, **b** distal bone segment in planned position, showing predrilled holes for screw fixation, **c** custom titanium plate with porous defect-filling augment for realignment and fixation

A high-resolution computed tomographic (CT) scan at routine clinical dosage was made of both forearms for preoperative planning (Philips Brilliance 64 CT scanner, Cleveland, OH; voxel size 0.45 × 0.45 × 0.45 mm, 120 kV, 150 mAs, pitch 0.6). The affected left and mirrored healthy right radii were segmented and proximally aligned to visualize the malunion (Fig. 2a). An anatomical coordinate system (Fig. 2a) was defined to quantify the deformity. The affected radius was shortened (9.9 mm). It also showed dorsal and radial collapse (31.4° and 8.8°), and rotational deformation (3.7°). These rotations revolve around the three axes of the anatomical coordinate system (*x*, *y* and *z*, respectively). Distal and proximal segments, excluding the deformity, were subsequently aligned with the mirrored image of the contralateral bone by registration [9, 10], to find the right anatomical alignment. Next, the position of the distal radius was corrected for bilateral length differences, to restore a normal ulnar variance. This quantification defined the complete relative position of the distal and proximal segments (Fig. 3b). Correcting the relative bone position required translations in the radioulnar, dorsopalmar and proximodistal direction of 4.8, 12.5 and 9.9 mm, respectively. Dorsopalmar flexion, radioulnar deviation and supination–pronation rotation required angular corrections of 31.4°, 8.8° and 3.7°.

To be able to bring the distal radius segment to the planned position, we first conducted a simulation using a patient-tailored plate [10], in combination with a porous defect-filling augment to fill the osteotomy gap and to provide additional mechanical support. To use the custom plate, predrilling for screw fixation and cutting the bone at the planned position is required. This is achieved using a drilling and cutting guide (Fig. 3a), which is tightly fitted to the patient’s own bone geometry. After application of the drilling and cutting guide, the plate and augment can be used to restore anatomical alignment of the distal and proximal segments (Fig. 3c). Position planning was performed using custom software [10].

To transfer the simulated virtual plan to the patient, a polyamide drilling and cutting guide, and a titanium plate and mesh (average porosity 70 %, average pore size 720 μm, thickness of solid struts ~350 μm) were created using additive manufacturing technologies. Guide and implant design and production were outsourced (Mobelife N.V., Leuven, Belgium).

### Surgical procedure

**Fig. 4:**
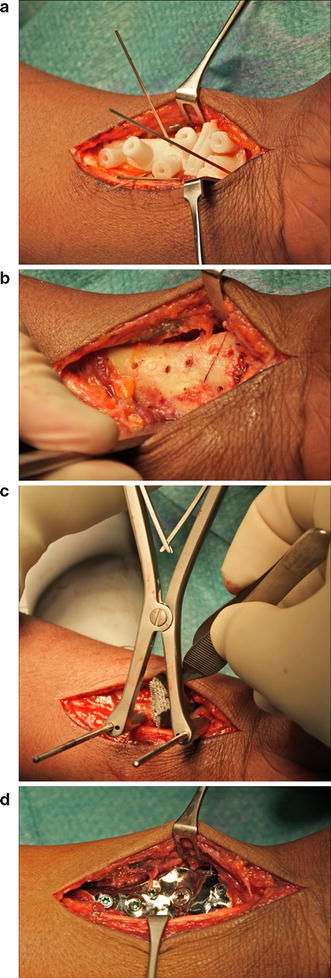
Surgical procedure showing, **a** fixation of the polyamide drilling and cutting guide using K-wires, **b** result of predrilling and partial cutting through guide slit. **c** Insertion of porous titanium mesh. **d** Mesh mounted to custom plate, custom plate fixated to bone using locking screws

After a volar approach of the distal radius, the polyamide guide tightly fit the bone. It was temporarily fixated using 1-mm Kirschner wires (Fig. 4a) before drilling screw holes (1.8 mm) and partial cutting of the bone using an oscillating surgical saw (blade thickness 0.65 mm) (Fig. 4b) through a slit in the guide. The osteotomy was completed by continuing the cut in the indicated direction. Next, two 4-mm K-wires were inserted for bone distraction (Fig. 4c) to enable inserting the titanium mesh into the osteotomy gap. The mesh was subsequently mounted to the custom plate using a single 3-mm titanium screw, and the plate was fixated to the bone using 2.4-mm locking screws [11] (Fig. 4d), to achieve reduction.

Postoperatively, the patient was encouraged to exercise her fingers, but full pro- and supination was not yet allowed. In this period, no complications occurred. After cast removal, the patient started to mobilize her wrist under physiotherapeutic guidance.

Physical examination after a follow-up period of 20 months showed wrist extension and flexion of both 60°. Radial and ulnar deviation were 10° and 30°. Forearm supination and pronation improved to 70° and 60°. The patient was satisfied about the renewed range of motion, but had complaints about pain of the scar and surrounding tissue, which is a common finding after performing wrist surgery.

**Fig. 5:**
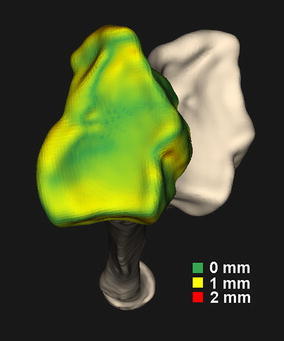
Patient radius showing malalignment of the distal segment (*off*-*white*) and the achieved position after corrective surgery (*color*-*coded segment*). The *colors* represent the local deviation from the planned position. This local deviation is defined as the shortest distance to the surface of the distal radius in the planned position (color figure online)

**Fig. 6:**
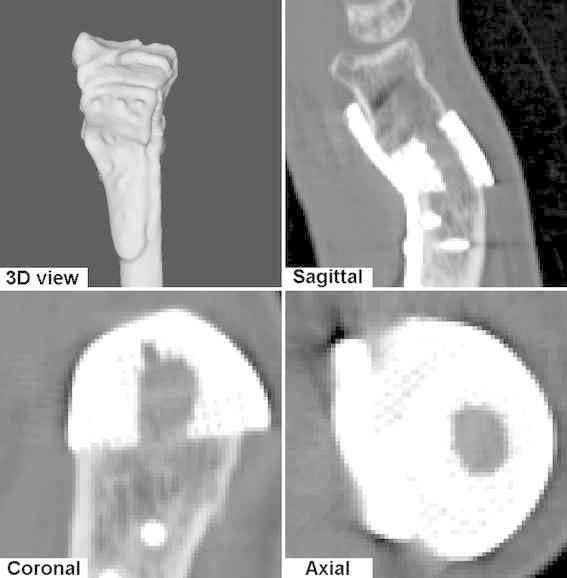
Follow-up (20 months postoperatively) 3D rendering of radius and custom plate, and multi-planar reconstruction showing osteointegration of the titanium mesh

A follow-up CT scan (20 months postoperatively) was made for quantitative evaluation of bone alignment and to determine whether osteointegration of the titanium mesh had occurred. Residual translations in the radioulnar, dorsopalmar and proximodistal direction were −1.2, 0.4 and 0.4 mm. Residual errors for dorsopalmar flexion, radioulnar deviation and supination–pronation rotations were −0.9°, −2.3° and 1.7°. This positioning error translated to a deviation of the distal running surface as depicted by Fig. 5, which shows a color map representing the local distance to the running surface of the distal radius segment in the planned position. Figure 6 shows a 3D surface rendering of the segmented radius, and a multi-planar reconstruction, which confirms osteointegration of the titanium mesh.

## Discussion

Conventionally, surgical planning is based on anteroposterior and lateral radiographs, which provide the radial inclination, the volar tilt and the ulnar variance [3]. Positioning is then achieved by subjectively bending a standard plate and subsequent screw fixation. This method has shown to be inaccurate [1] with residual translation and angulation errors up to 10 mm and 25°. Anatomical plates are assumed to fit the natural bone anatomy. However, it has been shown that subjective positioning of an anatomical plate may lead to considerable residual errors in individual patients [2]. Volar distal radius plate shapes further differ among manufacturers. One type may therefore fit better in a specific individual than another [2]. Moreover, anatomical plates generally do not follow the geometry of a *deformed* bone [10]. Accurate realignment of bone segments is nevertheless of the utmost importance since a statistically significant relationship has been shown between malpositioning and clinical outcome [1].

In the last decade, 3D virtual planning techniques are being used [3–8] because planning and postoperative evaluation based on radiographs is error prone since it hides rotations of the distal segment about the bone axis. Techniques to transfer the 3D virtual plan to the patient have been reported using free-hand navigated bone cutting and predrilling of screw holes [3, 5]. Other techniques help the surgeon in orienting the surgical saw or drill holes using patient-specific guides [6–8]. Before permanent fixation, for example by using a plate, bone segments have to be positioned in the right relative alignment. Techniques for temporary bone positioning in six degrees of freedom (three translations along, and three rotations about three orthogonal axes) have been proposed using optical tracking systems [3, 5] or passive clamping [9]. A disadvantage of these techniques is their heavy reliance on intraoperative imaging techniques, such as optical instrument tracking or intraoperative cone-beam CT imaging for patient-to-image registration. Thus, the operative procedure is relatively complex. In contrast, our proposed procedure using a patient-specific plate can be prepared entirely on a preoperative bilateral CT scan of the patient’s radii. As a result, no complex navigation instruments are required, and the surgical procedure is relatively simple. As shown in an experimental study [10] and in this patient case, accurate positioning can be achieved.

Our technique also has limitations. It does not enable deviations from the preoperative plan, including cases where the required soft tissue distraction appears to be impossible. Compared to standard osteosynthesis, the custom plate is also an order of magnitude more expensive. The higher costs for custom treatment include printing the cutting and drilling guide, printing the custom titanium plate and augment, adding threats to the plate for use with locking screws and adding a threat to the augment in order to mount it to the plate. The manufacturing costs may decline once metal printing techniques become more widely available. The proven relation between malalignment and reduced clinical outcome [11] may be a reason to spend effort in accurate surgical techniques instead of timely and costly aftercare. We believe that the advanced positioning characteristics of the custom plate will finally outweigh the current limitations. Future research on a larger patient group is needed to validate the therapeutic potential of customized plates.
